# Barrier Properties of GnP–PA-Extruded Films

**DOI:** 10.3390/polym12030669

**Published:** 2020-03-17

**Authors:** Regine Boldt, Andreas Leuteritz, Daniela Schob, Matthias Ziegenhorn, Udo Wagenknecht

**Affiliations:** 1 Leibniz Institute of Polymer Research Dresden (IPF), Hohe Strasse 6, 01069 Dresden, Germany; leuteritz@ipfdd.de (A.L.); wagenknt@ipfdd.de (U.W.); 2 Faculty Mechanical Engineering, Electrical and Energy Systems, Institute of Mechanical Engineering and Management, Brandenburg University of Technology Cottbus-Senftenberg, Universitaetsplatz 1, 01968 Senftenberg, Germany; daniela.schob@b-tu.de (D.S.); matthias.ziegenhorn@b-tu.de (M.Z.)

**Keywords:** graphene nanoplatelets, polyamide, nanocomposites, flat film extrusion, permeation rate

## Abstract

It is generally known that significant improvements in the properties of nanocomposites can be achieved with graphene types currently commercially available. However, so far this is only possible on a laboratory scale. Thus, the aim of this study was to transfer results from laboratory scale experiments to industrial processes. Therefore, nanocomposites based on polyamide (PA) and graphene nanoplatelets (GnP) were prepared in order to produce membranes with improved gas barrier properties, which are characterized by reduced permeation rates of helium. First, nanocomposites were prepared with different amounts of commercial availably graphene nanoplatelets using a semi-industrial-scale compounder. Subsequently, films were produced by compression molding at different temperatures, as well as by flat film extrusion. The extruded films were annealed at different temperatures and durations. In order to investigate the effect of thermal treatment on barrier properties in correlation to thermal, structural, and morphological properties, the films were characterized by differential scanning calorimetry (DSC), wide angle X-ray scattering (WAXS), optical microscopy (OM), transmission electron microscopy (TEM), melt rheology measurements, and permeation measurements. In addition to structural characterization, mechanical properties were investigated. The results demonstrate that the permeation rate is strongly influenced by the processing conditions and the filler content. If the filler content is increased, the permeation rate is reduced. The annealing process can further enhance this effect.

## 1. Introduction

Due to their layered structure and high aspect ratio, graphene nanoplatelets (GnP) and graphene oxide (GO) offer extraordinarily good possibilities for enhancing the barrier properties of polymer materials [1,2,3,4,5,6]. Many reports show that exfoliated and well oriented nanoplatelets reduce the permeability of gaseous molecules due to an elongated “tortuous pathway” [7,8,9,10,11,12]. This effect was even more pronounced when the filler content increases, provided that the fillers are homogeneously dispersed. Still, the majority of the studies handle (very) small amounts of graphene-like materials in the range of mg to prepare nanocomposites [9,13,14,15] due to the lack of procedures and shortage of suppliers capable of supplying amounts from 100 g to the kg range. This is necessary for investigation of graphene nanocomposite preparation in a scale adaptable to commercial processes, like compounding on a twin screw extruder and film extrusion. A survey on possible structures derived in polycarbonate with GnP available in an industrial scale is given by Poetschke and coworkers [16]. Another report uses graphene-like materials in high-density Polyethylene (HDPE) on large scale compounding machinery in a level up to 31%, indicating a behavior [17] comparable to classical fillers like talc [18], showing an increase of more than 300% at a comparable filler content of 35%. A study by Edelmann and coworkers [19] came to the conclusion that the processing of graphene-like materials on industrial like machinery does not lead to comparable results found on a lab scale. Our earlier studies emphasize these results [20]. Thus, the greatest challenge is still to obtain well dispersed graphene nanoplatelets. A typical method for the preparation of nanocomposites is melt compounding with highly shearing equipment twin screw extruders. In this way, GnPs are mixed directly with the polymer in the molten state and no solvents are required, making it an economical and environmentally friendly method for the mass production of nanocomposites. Another challenge is the bulk density of graphene nanoplatelets as a powder. The low apparent density of commercially available GnP powders leads to complications in extruder feeding [19].

The incorporation of nano additives has a significant effect on crystallization behavior. In particular, reports of GnP showing enhanced crystallinity can be found for polypropylene [21] and polyamide-6 [22]. Furthermore, it is known that crystallinity has beneficial effects on the permeation rate, where two aspects of crystallinity have to be considered: degree of crystallization and crystalline structure [23,24].

Considering these aspects, the objective of the present work is the development of a method for the incorporation of graphene nanoplatelets into the semi-crystalline polymer matrix by means of melt mixing.

Secondly, the barrier properties will be investigated as a function of filler content and crystallinity. Therefore, different amounts of commercially available graphene nanoplatelets were compounded, and GnP–polyamide (PA) nanocomposites films were prepared using flat film extrusion. For equilibration of crystallinity, the films were subsequently annealed at different temperatures and durations, leading explicitly to better barrier properties. In addition to improved barrier properties, reinforced polyamide can be expected as a result of incorporated graphene nanoplatelets.

## 2. Materials and Methods

### 2.1. Materials

Semi-aromatic polyamide polyarylamide (Ixef^®^ BXT 2000-0203, density: 1.12 g/cm^3^; MFI: 6 g/10 min at 240 °C) was kindly provided by Solvay, Brussels, Belgium. The graphene nanoplatelets EXG 98 300R (diameter: 24 µm, surface area: 300 m^2^/g) with lamellar structure were supplied from Graphit Kropfmühl/AMG Mining AG, Hauzenberg, Germany. Ultramid^®^ B27E from BASF, Ludwigshafen, Germany and Cloisite^®^ 30B from Southern Clay Product, Gonzales, TX, USA, were used as dispersants.

### 2.2. Preparation

The nanocomposites were prepared in a three-step method. In the first step, EXG 98 300R and Cloisite^®^ 30B (ratio 1:4) were mixed in H_2_O/Isopropanol under continuous stirring following the procedure described in [25]. Then, the paste was dried until constant weight. In the second step, a masterbatch of EXG 98 300R/Cloisite^®^ 30B and Ultramid^®^ B27 (ratio 3:7) was prepared by melt compounding, using a corotating intermeshing twin screw extruder ZE25 (KraussMaffei Berstorff, Hannover, Germany) at 270 °C with a rotor speed of 300 rpm and a flow rate of 10 kg/h. In the third step, the masterbatch was diluted to the desired concentration by compounding with Ixef^®^ BXT 2000-0203 using the same parameters as used for preparation of masterbatch.

Subsequently, films with a thickness of 200 µm were produced by compression molding at 240 °C and 270 °C (50 kN) using a hot press (Model PW 40 EH, Paul Otto Weber GmbH, Remshalden, Germany). Additionally, films with a thickness of approximately 300 µm were prepared by film extrusion using an Extruder equipped with a flat film die and a Calander (Vario G 168/400, Dr. Collin GmbH, Germany). A temperature profile of 270 °C to 240 °C from hopper to die was used in flat film extrusion. The temperature of the rolls was set to 25 °C. For annealing, the extruded films were heated under vacuum at 80 °C and 120 °C for 1 h, and at 120 °C for 1, 2 and 4 h and cooled down slowly.

Initials characterizing the processing conditions give the sample designation of produced films (Table 1). Thus, CM means compression molding, and ME stands for flat film extrusion. The letter *x* stands for filler content, which is 0, 1.5, 3.0 and 6.0 wt. percentage. The last number indicates the temperature at which the film was compression molded or annealed.

### 2.3. Characterization

The degree of filler dispersion in the nanocomposites was characterized by transmission electron microscopy (TEM) and rheological measurements. TEM images were performed on a Zeiss Libra200 MS (Carl Zeiss GmbH, Oberkochen, Germany) with an accelerating voltage of 200 kV. The TEM samples (ultrathin slices, with a thickness of 50 nm) were cut from films perpendicular to the extrusion direction and compression mold direction. The specimens were prepared with a diamond knife (DIATOME Ltd, Nidau, Switzerland) at −120 °C using a cryo-Ultramicrotome UC7 (Leica Mikrosysteme Vertrieb GmbH, Wetzlar, Germany).

Melt rheological measurements were performed in the linear viscoelastic domain using an ARES rheometer (Rheometrics Scientific, New Castle, DE, USA) in oscillatory mode and plate–plate geometry with 25 mm and 1 mm gap size. Before measurement, all samples were dried for 4 h at 85 °C in an oven under vacuum conditions to prevent moisture induced degradation phenomena. The rheological measurements were performed in a nitrogen atmosphere at 270 °C at a constant strain amplitude of 1%, which proved to ensure linear viscoelasticity during the measurements. The angular frequency was increased logarithmically from 0.05 to 100 rad/s. To estimate the degree of dispersion, shear thinning exponents (n) were calculated according to Wagener [26] by applying the following equation:

(1) $$\eta =A\omega^{\left(n\right)}$$

The degree of crystallization was evaluated by comparing the enthalpies of the first heating cycle from the DSC measurements. The measurements were performed with approximately 5 mg of the samples under nitrogen in a temperature range from −60 °C to 300 °C, with a heating rate of 10 K/min using a Q2000 (TA Instruments, New Castle, DE, USA).

The crystal structure as a function of filler was examined using a wide-angle X-ray diffractometer Bruker D8 Advance (Bruker AXS GmbH, Karlsruhe, Germany) from 2Θ = 5° to 50° at room temperature. To evaluate structural changes during the reheating process, X-ray diffractograms were recorded at different temperatures using a multi-range device, Ganesha 300 XL+ (SAXSLAB ApS, Kongens Lyngby, Denmark), with CuKα radiation. The path of rays, sample, and detector are completely under vacuum (p < 0.05 mbar).

The crystal morphology was investigated by using a polarized optical microscope Axioimager.A1m (Zeiss, Berlin, Germany), combined with a camera (Axiocam ICc3) and the software Axio vs. 4.8.1.0 for capturing the images. The samples were prepared under cryo conditions. Specimens with a thickness of 10 µm were cut using a microtome Leica RM2265 (Leica Mikrosysteme Vertrieb GmbH, Wetzlar, Germany).

Tensile tests were performed on dumbbell-shaped tensile test specimens using a universal testing machine, InspekTable 10 kN (Hegewald & Peschke, Nossen, Germany). The samples were punched with extruded films parallel and perpendicular to the flow direction. For each analyzed system, five valid samples were tested at room temperature with a constant cross-head speed of 1 mm/min.

Permeation measurements of helium were applied at room temperature using a GDP-C (Brugger, München, Germany). The permeability of helium was calculated from measured gaseous transmission rate (GTR) for film with a thickness of 300 µm. At least two permeation measurements were performed for each sample. The mean value, including standard deviation, is given in the results. In case of high deviations, one or two additional permeation measurements were carried out.

## 3. Results

Due to the very low apparent density of GnP, a method of combining the GnP with the dispersant C30B was established. Organomodified clays could be easily dispersed in a mixture of isopropanol and water. Through the subsequent addition of GnP and clay, a dense paste was finally derived, which could be used for conventional processing, i.e., drying and grinding. The derived powder was free flowing and well suited for gravimetric dosing into the hopper of the twin screw extruder for preparation of the masterbatch. The masterbatch was subsequently diluted to the desired level of filler. For the dilution step, a semi-aromatic polyamide with low processing temperature was selected. As expected, due to the interaction of the aromatic structure with the graphene nanoplatelets, a better dispersion and distribution could be derived. The aim of the addition of clay, which is extraordinarily dispersible in PA-6, is to support the shear load on the GnP during processing. Clays are well known as a viscosity modifier at low loadings. Furthermore, the used clay has a reported size of approximately 100–150 nm [27]. Thus, its contribution to a reduction of permeability is small against the contribution of the GnP with a lateral dimension of 20 µm, according to the modified Nielson model [7,28].

**Figure 1:**
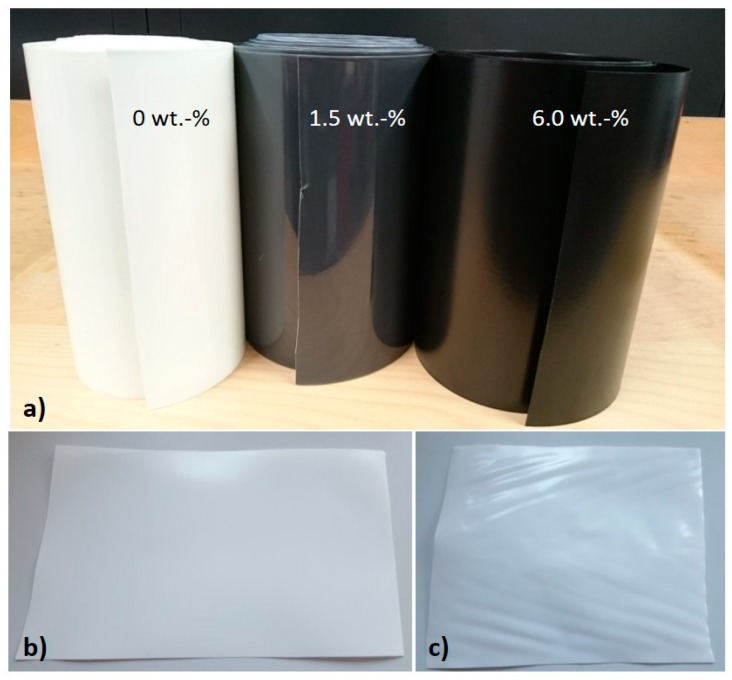
Rolls of extruded films with different filler content. (**a**) A piece of extruded film with 0 wt.-% filler before thermal treatment, and (**b**) after thermal treatment at 120 °C (**c**).

Films were prepared by flat film extrusion and are presented in Figure 1a. The films show homogenous and very smooth surfaces. Pieces of films were annealed in order to investigate the effect of thermal treatment on barrier properties. Before thermal treatment, the films appear flat (Figure 1b); while after thermal treatment, warpages can be observed. This effect is particularly pronounced at the edges of the films along the flow direction (Figure 1c).

### 3.1. Rheological Properties

**Figure 2:**
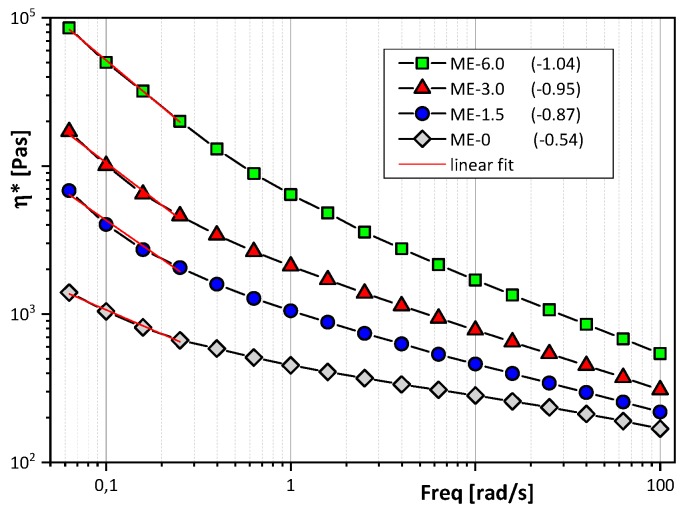
Complex viscosity with different filler content and shear thinning exponents given in brackets.

In order to characterize the influence of filler content on the main viscoelastic parameters, the complex viscosity (η*) has been measured at 270 °C as a function of frequency. In the studied frequency range, it is obvious that all samples show shear thinning behavior, which is attributed to reduced entanglements density under the influence of high shear stress (Figure 2). Furthermore, it can be observed that the complex viscosity increases with increased filler content, due to the enhanced interaction between the filler surface and polymer melt. In order to evaluate the quality of filler dispersion, shear-thinning exponents were calculated as slopes of complex viscosity at low shear rates. The fitting curves are given in Figure 2 (red lines), as well as the shear thinning exponents (data in brackets) for samples with different amount of filler. With increased filler content, the shear-thinning exponent increases as an indication of improved filler dispersion.

### 3.2. Filler Dispersion

**Figure 3:**
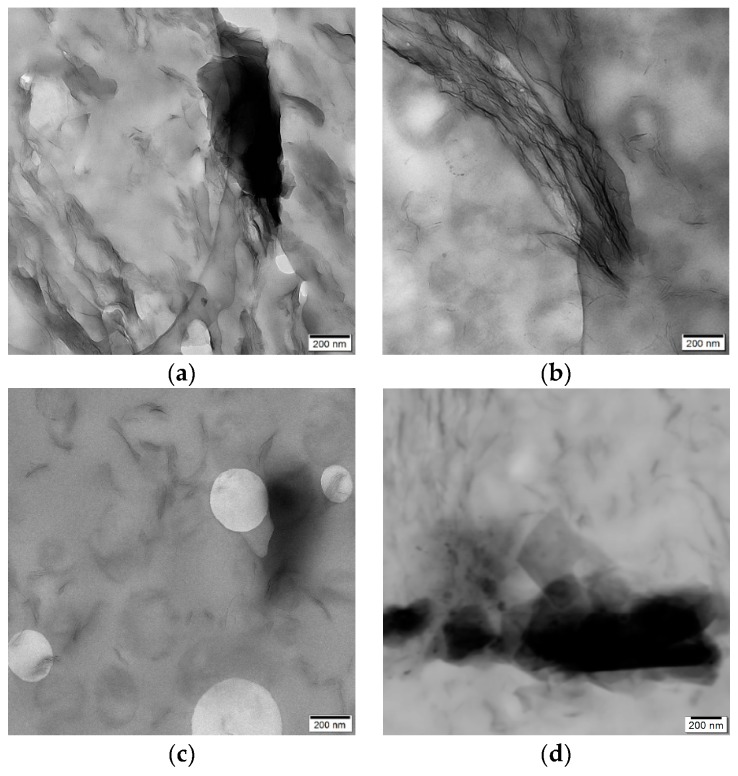
TEM images of ultrathin sections from films based on nanocomposites with 3.0 wt.-% filler: (**a**) CM-3.0-240, (**b**) CM-3.0-270, (**c**) ME-3.0, and (**d**) from granule with 3.0 wt.-% filler before preparation of films.

The morphology in regard to filler distribution and filler dispersion is presented in Figure 3 for representative samples with 3.0 wt.-% filler content. In the compression molded samples (Figure 3a,b), large aggregates of graphene nanoplatelets are dominant. However, individual platelets stacked in a loose connection can also be seen.

The same morphology can be observed in the nanocomposites before compression molding (Figure 3d), showing that the presence of GnP aggregates in the compression molded films is not caused by reagglomeration.

**Figure 4:**
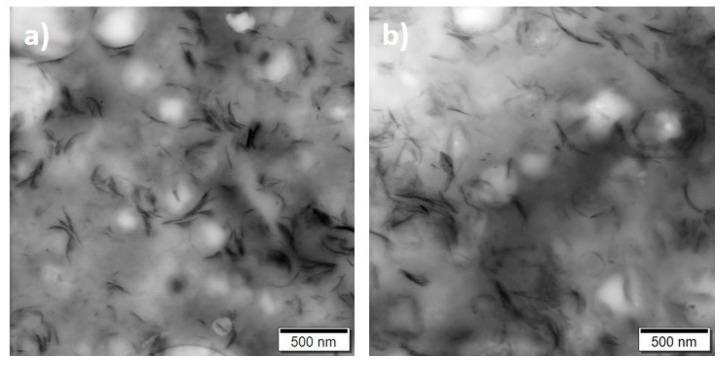
TEM images of extruded films (**a**) before thermal treatment (ME-3.0) and (**b**) after thermal treatment ME-3.0-120.

Smaller aggregates of graphene nanoplatelets and more separated fillers can be observed in the melt-extruded samples (Figure 3c). This morphology doesn’t change after thermal treatment of extruded films. Well distributed fillers can be seen in the samples before and after thermal treatment (Figure 4), from which it can be concluded that the thermal treatment has no influence on the filler distribution.

In contrast, the processing conditions of films affects the quality of filler dispersion significantly. From comparison of filler dispersion in compression molded samples and extruded films (Figure 3), it can be assumed that additional shear forces during film extrusion will improve the dispersion of GnP within the polymer matrix, compared to compression molded samples where no additional shear forces occur.

### 3.3. Thermal Properties

**Figure 5:**
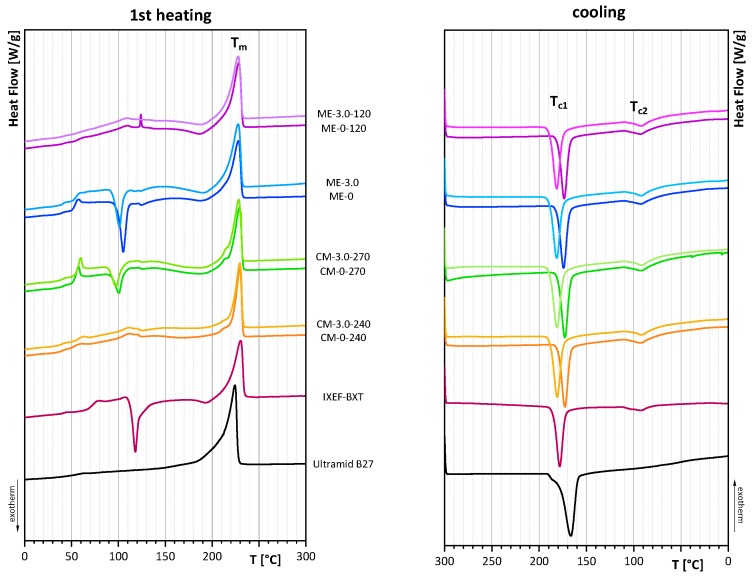
Differential scanning calorimetry (DSC) curves of the first heating cycle (**left**) and of cooling (**right**) of virgin polymers and films with 0 wt.-% and 3.0 wt.-% filler content.

DSC experiments were performed in order to analyze the effect of filler, processing conditions, and thermal treatment on the crystallization behavior. Comparison of melting curves and cooling curves of virgin polyamides (IXEF^®^BXT, Ultramid B27) and samples with 0 wt.-% and 3.0 wt.-% filler content are presented in Figure 5.

The corresponding melting temperature (T_m_) of the first heating cycle, as well as the melting enthalpies (ΔH_m_) and the crystallization temperatures (T_c1_ and T_c2_), are given in Table 2.

**Figure 6:**
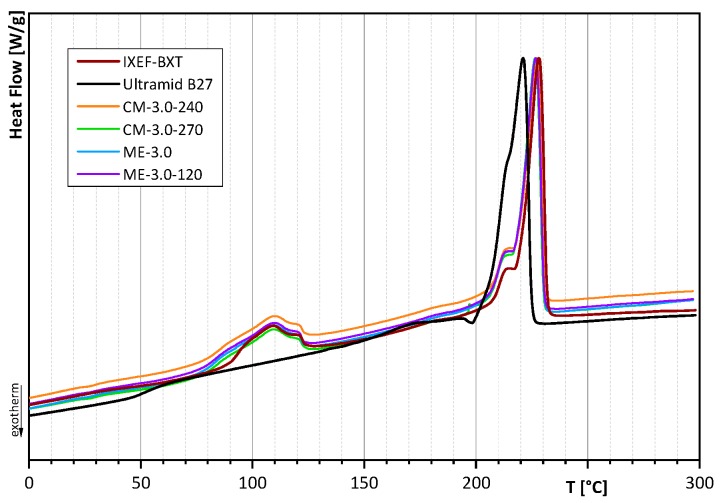
DSC curves of the second heating cycle of untreated polymers and of films with 3.0 wt.-% filler.

The melting curves of the first heating cycle show similar melting points at about 229.9 ± 0.6 °C for all compression molded samples, which is identical to the melting point of untreated IXEF^®^BXT at 230.3 °C. The melting point of Ultramid B27 was determined at 223.6 °C. In melt-extruded films, a slight shift towards lower melting temperatures is observed. The melting points of melt-extruded films are 227.9 ± 0.4 °C. This effect can be attributed to different cooling conditions after film preparation and was confirmed by the analysis of the melting point after the second heating cycle (Figure 6), where all samples have the same thermal history. The melting point of the second heating cycle was calculated to be 226.6 ± 0.3 °C for all samples, regardless of processing conditions or filler content. The only exception is Ultramid B27 with a melting point of 220.9 °C. It should be noted that all melting curves of the second heating cycle as characteristics for material properties are identical with respect to peak position (melting point) and peak shape, with the exception of Ultramid B27. However, since no additional peak occurs, this result indicates a homogeneous miscible blend without phase separation.

The melting curves of the first heating cycles also show that the DSC curves of samples prepared by compression molding at 270 °C (CM-0-270, CM-3.0-270) and flat film extrusion (ME-0, ME-3.0) show a typical peak for cold crystallization at about 105 °C. This can be attributed to rapid cooling, which suppresses crystallization. Therefore, the enthalpy of the first heating cycle is much lower for rapidly cooled samples, than for slowly cooled samples. Slowly cooled samples have sufficient time for crystallization, so that the peak for cold crystallization does not appear in the DSC curves, and much higher enthalpies have been estimated. This can be seen in samples that were compression molded at 240 °C (CM-0-240, CM-3.0-240) and in extruded films that were annealed at 120 °C (ME-0-120, ME-3.0-120).

In contrast to the melting point, the crystallization temperature will be influenced by the filler content. The crystallization temperature (T_c1_) of samples with 3.0 wt.-% filler is significantly higher, compared to the crystallization temperature of samples without filler. Films without filler crystallize at approximately 173.3 ± 0.6 °C, while films with 3.0 wt.-% filler content crystallize at 181.2 ± 0.2 °C. That means that the fillers act as nucleating agents.

### 3.4. Crystal Structure and Morphology

Wide angle X-ray scattering (WAXS) and optical microscopy were used to verify the crystal structure and crystal morphology of extruded films before and after thermal treatment.

**Figure 7:**
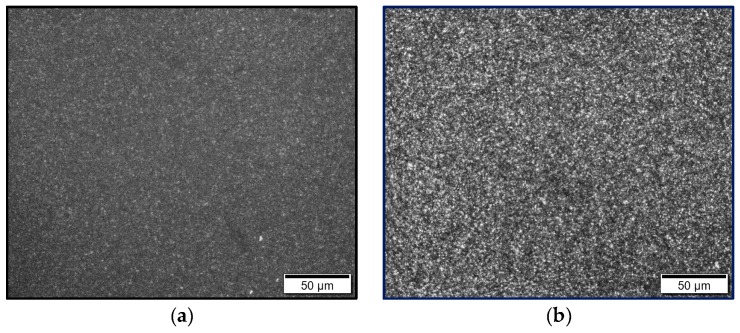
Optical microscopy (OM) images of extruded films ME-0 (**a**) before thermal treatment and (**b**) after thermal treatment.

In accordance to the results of DSC measurements, different morphologies could be identified by polarization microscopy (Figure 7). Prior to thermal treatment, crystalline structures are hardly visible (Figure 7a) because of rapid cooling conditions. Crystalline structures are formed only by thermal post-treatment with subsequent slow cooling rates. Therefore, crystalline superstructures in the form of small spherulites are only visible after reheating (Figure 7b).

Because the thermal treatment is performed below the melting point, it can be assumed that during melt extrusion, a large number of crystal nuclei are formed which grow to spherulites during annealing.

**Figure 8:**
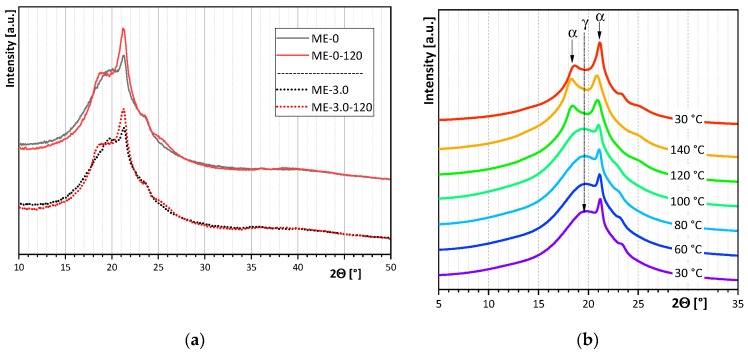
Wide angle X-ray scattering (WAXS) curves of (**a**) melt-extruded films as a function of different filler amounts and thermal treatment, (**b**) ME-0 at different temperatures.

Figure 8 shows the WAXS patterns of melt extruded films as a function of different amounts of filler (Figure 8a) and thermal treatment (Figure 8a,b). The solid lines in Figure 8a represent the diffractograms of films without filler, while the dotted lines represent the diffractograms of melt-extruded films with 3.0 wt.-% filler. It can be observed that the addition of filler does not influence the crystallization. The diffractograms of films with and without filler are identical. This applies both to samples before thermal treatment (black lines) and to samples after annealing (red lines). However, it is obvious that thermal treatment plays a crucial role in crystallization. WAXS patterns of films before thermal treatment differ from patterns of films after thermal treatment. Before thermal treatment, a mixture of α-phase and γ-mesophase is observed. After thermal treatment, mainly the α-phase is observed. The transformation from the γ-mesophase into the α-phase was recorded with WAXS at different temperatures (Figure 8b). During annealing, the γ-mesophase disappeared at a temperature of 120 °C, and a fully α-phase was obtained.

### 3.5. Mechanical Properties

**Figure 9:**
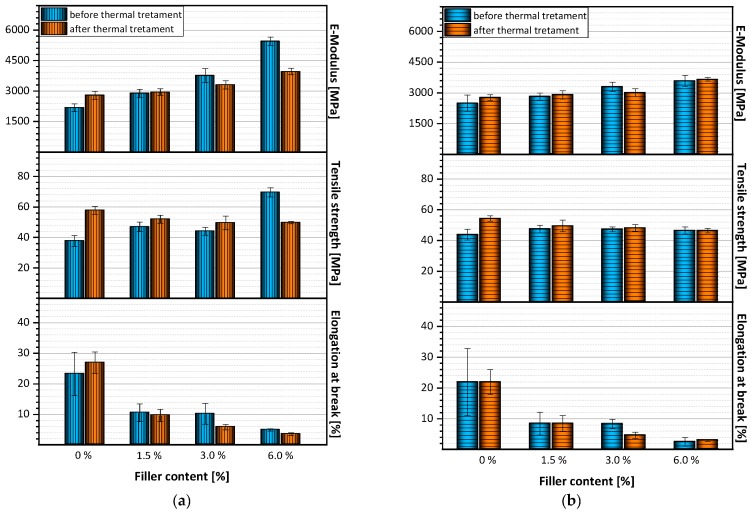
Mechanical properties of melt-extruded films before and after thermal treatment (**a**) in flow direction, (**b**) perpendicular to flow direction.

Figure 9 shows the mechanical properties of melt-extruded films before and after thermal treatment as a function of filler quantity. It can be seen that elongation at break apparently decreases with the addition of fillers. Films without filler achieve an elongation at break of approximately 23.7%, which is increased to 26.9% when the film is annealed to 120 °C. The elongation at break is reduced to approximately 10% with the addition of 1.5 wt.-% and 3.0 wt.-% filler, and to 4.9% when 6.0 wt.-% filler is added. The same trend can be observed after thermal treatment of the films.

In contrast to reduced elongation at break, modulus and tensile strength increase with increasing filler content. The modulus of pure PA films is approximately 2180 MPa, and increases to approximately 5450 MPa when 6.0 wt.-% filler is added. Similar behavior can be observed after thermal treatment, whereby the maximum modulus value for films with 6.0 wt.-% filler is only 3960 MPa.

As far as the effect of thermal treatment is concerned, the tensile strength shows a rather unusual behavior. Before thermal treatment, the tensile strength increases with increasing filler content. Films with 6.0 wt.-% filler achieved values of approximately 69.9 MPa, which is significantly higher than the tensile strength of pure PA films with approximately 37.7 MPa. This effect was to be expected. It indicates reinforcement by good filler dispersion within the polymer matrix. It was therefore even more surprising to observe the reverse trend in tensile strength after annealing the films. After thermal treatment, the tensile strength of pure PA films is approximately 57.7 MPA, while the tensile strength of films with 6.0 wt.-% filler only reached 49.7 Ma.

At this point, it has to be mentioned that the results discussed above were measured on samples perpendicular to the direction of flow (Figure 9a). It is interesting to note that in specimens prepared parallel to the flow direction, identical behavior was observed with respect to values for E-modulus, tensile strength, and elongation at break (Figure 9b). These results show that mechanical properties of films produced by flat film extrusion are not affected by the flow direction.

### 3.6. Permeation

**Figure 10:**
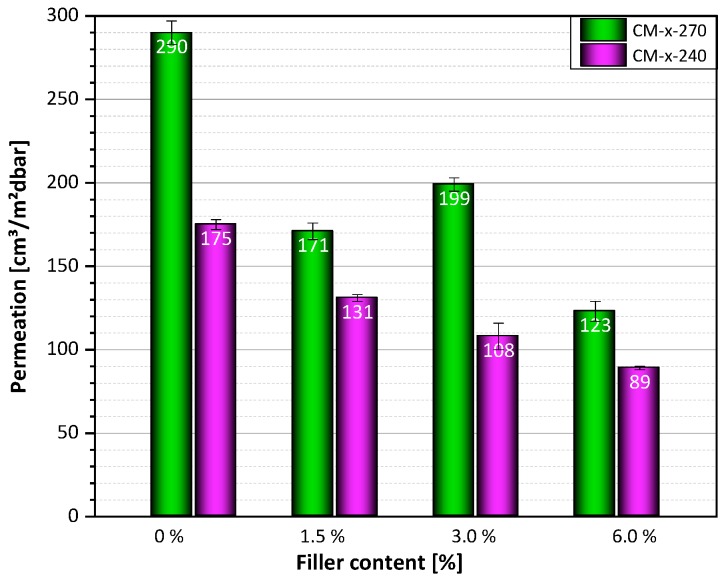
Permeation rates of films with different filler content compression-molded at 240 °C and 270 °C.

**Figure 11:**
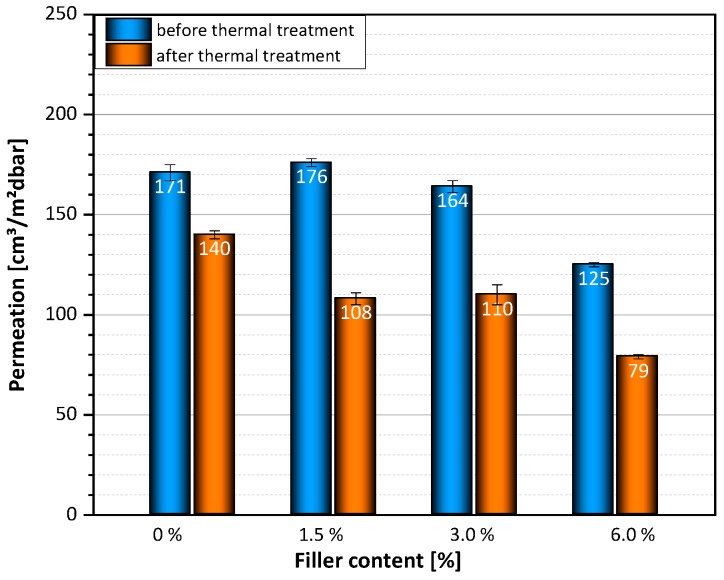
Permeation rates of melt-extruded films with different amount of fillers before and after thermal treatment.

In accordance to the theory, the permeation rate decreases with increasing filler content. This was shown for both compression-molded plates (Figure 10) and extruded films (Figure 11). For samples compression-molded at 270 °C, a reduction of the permeation rate from 290 cm^3^/m^2^dbar for samples without filler to 123 cm^3^/m^2^dbar for samples with 6.0 wt.-% filler content was observed. The same trend, but with significantly lower permeation rates, was achieved when the samples were compression-molded at 240 °C. The permeation rate was reduced from 175 cm^3^/m^2^dbar for unfilled samples to 89 cm^3^/m^2^dbar for samples with 6.0 wt.-% filler content. This effect can be explained by the different degree of crystallization caused by different cooling rates. Slower cooling rates lead to a higher degree of crystallization and reduced permeation rates.

Similar behavior was observed with extruded films. The permeation rates of GnP–PA-extruded films decrease with increasing amount of fillers. The permeation rate was reduced from 171 cm^3^/m^2^bar for unfilled samples to 125 cm^3^/m^2^bar for samples with 6.0 wt.-% filler content. In addition, the subsequent thermal treatment of the extruded films further reduced the helium permeability (Figure 11). After the thermal treatment, the permeation rate was reduced from 140 cm^3^/m^2^dbar for pure PA-films to 79 cm^3^/m^2^dbar for samples with 6.0 wt.-% filler. This effect can be attributed to a change in the degree of crystallization.

Finally, it should be mentioned that the permeation rate was not affected when the films were heated below 120 °C. An increase in the residence time also had no effect on helium permeability.

## 4. Conclusions

In the present work, nanocomposites based on polyamide and graphene nanoplatelets could be processed into films with improved gas barrier properties at laboratory scale, as well as at semi-industrial scale using flat film extrusion. The influence of filler content and processing conditions on barrier properties, by means of helium permeability, was evaluated. It was shown that the permeation rate was reduced with increased filler content. This effect can be enhanced by optimizing process conditions, with respect to the cooling rate. Rapid cooling rates lead to suppressed crystallization, while slow cooling lead to an increased degree of crystallization, which ultimately leads to an increased permeation rate. The best performance with respect to permeation rate was achieved in melt-extruded films after annealing.

## Figures and Tables

**Table 1:** Designation of prepared and investigated films.

Sample	Process	Sample Designation
compression molded film	pressed @ 240 °C	CM-*x*-240
	pressed @ 270 °C	CM-*x*-270
melt extruded film	without reheating	ME-*x*
	reheated @ 80 °C	ME-*x*-80
	reheated @ 120 °C	ME-*x*-120

**Table 2:** Melting temperatures (T_m_), crystallization enthalpies (ΔH_m_), and crystallization temperatures (T_c_) of virgin polymers and films with 0 wt.-% and 3.0 wt.-% filler.

	T_m_ [°C]	ΔH_m_ [J/g]	T_c1_ [°C]	T_c2_ [°C]
**Ultramid B27**	223.6	102.5	166.4	-
**IXEF^®^BXT**	230.3	11.1	178.2	92.0
**CM-0-240**	229.7	51.0	172.7	91.9
**CM-0-270**	229.2	36.1	172.7	91.3
**ME-0**	227.5	9.3	174.2	91.5
**ME-0-120**	228.5	37.8	173.5	91.8
**CM-3.0-240**	229.6	50.0	180.8	91.0
**CM-3.0-270**	230.9	25.2	181.0	91.0
**ME-3.0**	227.7	12.1	181.3	91.5
**ME-3.0-120**	227.9	40.0	181.5	91.7
